# The Use of ctDNA in the Diagnosis and Monitoring of Hepatocellular Carcinoma—Literature Review

**DOI:** 10.3390/ijms24119342

**Published:** 2023-05-26

**Authors:** Agnieszka Kopystecka, Rafał Patryn, Magdalena Leśniewska, Julia Budzyńska, Ilona Kozioł

**Affiliations:** 1 Students’ Scientific Circle on Medical Law, Department of Humanities and Social Medicine, Medical University of Lublin, 20-093 Lublin, Poland; aga.kop@interia.eu (A.K.); lesniewska.w.magdalena@gmail.com (M.L.);; 2 Department of Humanities and Social Medicine, Medical University of Lublin, 20-093 Lublin, Poland

**Keywords:** ctDNA, hepatocellular carcinoma, liquid biopsy

## Abstract

Hepatocellular carcinoma (HCC) is the most common primary liver cancer and is one of the leading causes of cancer-related deaths worldwide. Despite advances in medicine, it is still a cancer with a very poor prognosis. Both imaging and liver biopsy still have important limitations, especially in very small nodules and those which show atypical imaging features. In recent years, liquid biopsy and molecular analysis of tumor breakdown products have become an attractive source of new biomarkers. Patients with liver and biliary malignancies, including hepatocellular carcinoma (HCC), may greatly benefit from ctDNA testing. These patients are often diagnosed at an advanced stage of the disease, and relapses are common. Molecular analysis may indicate the best cancer treatment tailored to particular patients with specific tumor DNA mutations. Liquid biopsy is a minimally invasive technique that facilitates the early detection of cancer. This review summarizes the knowledge of ctDNA in liquid biopsy as an indicator for early diagnosis and monitoring of hepatocellular cancer.

## 1. Introduction

Hepatocellular carcinoma (HCC) is the sixth most commonly occurring cancer worldwide and, due to its constantly increasing incidence, has become the third leading cause of cancer-related death among general populations. Moreover, it represents the most common cause of death in patients with cirrhosis [1,2]. HCC is more common in men than in women [3,4]. There has been an increase in HCC incidence in recent years, a trend particularly seen in Sub-Saharan Africa, Eastern Asia, and the Southeast, primarily as a result of high HBV prevalence rates [5,6].

The definitive therapies for HCC remain surgical resection and liver transplantation that can be performed only in patients at very early (0) and early (A) stages. However, given the similar survival benefit paired with the less invasiveness and lower costs compared to surgical resection, percutaneous ablative therapies such as radiofrequency ablation (RFA) and microwave ablation (MWA) are now considered the first treatment approach in both very early and early stages [7]. 

Patients with hepatitis B or C (HBV or HCV), alcohol abuse or metabolic syndrome are predisposed to developing HCC [3,8,9,10,11]. Imaging, alpha-fetoprotein (AFP) levels, and tissue biopsy are the main methods used to detect and monitor relapses of HCC [9,12]. Despite the improvement in screening and surveillance programs, most patients with HCC (about 65–70%) are still diagnosed in the intermediate (B) or advanced (C) tumoral stages, thus resulting ineligible for radical therapies, treatment options for advanced disease are limited [8,13,14]; therefore, patients with intermediate and/or advanced HCCs are considered for transarterial therapies or systemic therapies which, albeit effective, are deemed non-curative or “palliative” and still yield a lower 5-year survival rate [9,15,16]. 

One of the keys to personalized treatment is gene profiling. Harvesting tissue samples via biopsy is an effective way to collect the right specimen for further analysis. However, liver biopsy is an invasive procedure. Biopsies often yield insufficient amounts of cancer cells and are not always available [17]. According to Renzulli et al., 2022 the use of liver biopsy is limited by a raised rate of false negative results (30%). Another limitation is an insufficient sampling rate of up to 15% [18]. The role of imaging studies in HCC diagnosis has a significant value, especially in cirrhotic patients. The diagnosis of HCC in this group of patients can be based on imaging findings alone, and treatment decisions can be made without tissue sampling. Despite the recent technological developments, including the use of hepato-specific contrast media in MRI, imaging diagnosis still has important limitations, especially in non-cirrhotic patients, in those with very small nodules (<1 cm), and in those who have nodules lacking the characteristic imaging features of HCC [19]. The serum concentration level of alpha-fetoprotein (AFP) is the most commonly used biomarker of HCC. However, the sensitivity and specificity of this compound are mediocre [20,21]. Furthermore, elevated AFP is associated more strongly with advanced stages of HCC than with early stages of the disease, thus questioning the efficacy of AFP measurement for early HCC screening [22,23].

Currently, no diagnostic method has 100% sensitivity and specificity and can be used in every patient. Commonly used markers used to detect HCC have poor diagnostic efficacy, while imaging and histopathology have limitations in diagnostic accuracy and sensitivity [9,24]. Therefore, it is very important to find an effective method for detecting HCC at an early stage of the disease and monitoring its recurrence.

## 2. Liquid Biopsy

In 1977, researchers first described circulating tumor DNA in human plasma, confirming that it contained mutations characteristic of cancerous cells [25,26,27,28].

**Figure 1:**
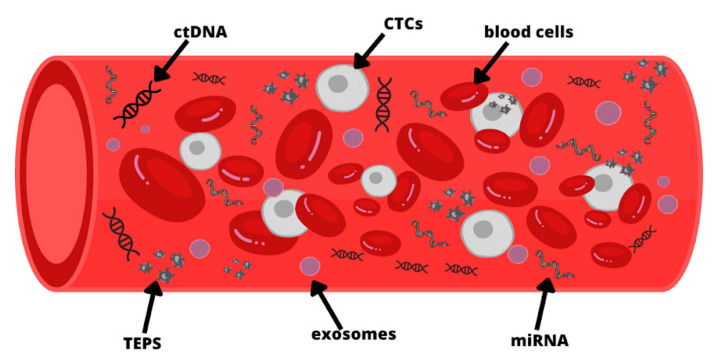
Molecules detected in peripheral blood for liquid biopsy: circulating tumor DNA (ctDNA), circulating tumor cells (CTCs), tumor-educated platelets (TEPS), microRNA (miRNA). The figure is only for illustrative purposes. There is no indication of the actual size of each element.

Liquid biopsy is the collection for analysis of a sample of unstable biological tissue of the body [9,29], and it is a minimally invasive technique that facilitates early detection of cancer [30,31]. Over the past few years, the development of molecular techniques enabled the detection of circulating tumor cells (CTCs) and cell-free DNA (ctDNA). Cancer patients present a higher level of ctDNA compared to healthy people. In addition to this, in a liquid biopsy, cancer patients also present cell-free microRNA and extracellular RNA, such as exosomes or tumor-educated platelets (TEPS) (Figure 1) Ref. [9]. Circulating tumor DNA (ctDNA) is tumor-specific DNA in the circulation released after metabolic secretion, apoptosis, or necrosis [17], and the circulating half-life of ctDNA is up to two hours [32]. The ctDNA provides dynamic, detailed information about tumor biology without the need for frequent biopsies [33,34]. Performing a liquid biopsy is particularly advantageous when the amount of tissue is too small to perform repeated tissue biopsies [3]. Serial sampling can be accomplished with minimal invasive sample collection [27]. Currently, research is underway to optimize ctDNA technology for use in clinical practice [17]. Combining cell-free DNA with AFP marker and age might be a promising tool in HCC diagnosis [35]. DNA methylation signatures, copy number aberrations, and somatic mutations have all been characterized to date in ctDNA [9,26,36,37,38,39]. Knowledge of genetic mutations affecting the development and progression of HCC allows for a better understanding of this disease [3]. The ctDNA can accurately classify tumor stage, dynamically adjust treatment plans, and plan surgical resection and postoperative therapy [33]. Performing liquid ctDNA biopsies can help monitor response to therapy, early detection of non-response and knowledge of tumor recurrence even months before clinical signs of recurrence [26,40,41].

Liquid biopsies require two 10 mL blood samples that together contain 5 ng of DNA for ctDNA analysis [8,11,42]. The physical characteristics and molecular characteristics of CTCs can be used to distinguish them from nucleated cells or normal epithelial cells, including electric charge, density, measurement, transfer capacity, and deformation ability, as well as biological parameters, such as cell surface markers, or a combination of both characteristics [43]. The methods of detection can be divided into the following groups: methods targeted to assay a few known mutations using PCR (digital PCR, amplification-refractory mutation system (ARMS)-PCR, etc.) and untargeted methods to sequence millions of DNA fragments (Sanger sequencing, next-generation sequencing (NGS), etc.) [44,45,46]. All of them must be highly sensitive and specific due to the very small amount of DNA material, which can be extracted from a 1 mL blood sample [47]. In addition to single-locus/multiplexed assays, targeted sequencing and genome-wide sequencing are available, depending on the size of the assay panel. Sequencing using PCR can be used for single loci and multiplexed analyses, and NGS can be applied to any size panel [45]. NGS plasma ctDNA, the so-called liquid biopsy, is an analytically very sensitive and specific test, detecting single molecules of tumor DNA, >85% of single nucleotide polymorphisms (SNPs) that are present in patients with advanced tumors, with analytical specificity > 99.9999% [48]. 

### 2.1. Early Diagnosis

The utility of ctDNA assessment may be particularly important in patients with suspected HCC, as these patients are often not biopsied due to potential complications [8]. The use of ctDNA in oncological diagnostics may, in the future, replace standard cancer markers such as AFP. The ctDNA is more specific because it is detected based on the genetic changes present in the cancer cells. Another advantage of ctDNA over AFP is that AFP is secreted in only 70% of HCC patients [5,40]. Patients with non-elevated AFP in their blood benefited most from the genomic aspects. The study detected 44 of 76 HCCs (57.9%) by elevating AFP, a widely used diagnostic biomarker for HCC. As a comparison, DNA detection of 14 additional HCCs (18.4%) was achieved by combining the genomic classifier copynumber aberrations and fragment size in patients whose HCC had been missed by AFP testing alone [6]. Wang J. et al., 2020 conducted a study of 81 patients with HCC undergoing hepatectomy. Peripheral blood samples were taken before and after surgery. In total, 70.4% (57/81) of them had detectable ctDNA prior to surgery, while the positive AFP rate was only 56.8%—the ctDNA diagnostic capability was better than AFP. Positive preoperative ctDNA status was associated with larger tumor size, multiple tumor lesions and Microvascular Invasion, advanced stage BCLC (Barcelona Clinic Liver Cancer), shorter Disease-Free Survival (DFS) and Overall Survival (OS). The analysis showed that ctDNA was an independent risk factor for postoperative recovery [49].

### 2.2. Guiding Personalized Therapy

The use of ctDNA liquid biopsy is very useful in the search for drug-sensitive gene markers and the identification of possible resistance mechanisms in cancer patients [50]. Mody K. et al., 2017 conducted a study in which 35 patients with HCC were subjected to ctDNA testing. It was found that ctDNA can be a very good non-tissue alternative to genomic profiling in patients with HCC [17]. The main advantage of ctDNA is believed to be its ability to store comprehensive somatic information about primary HCC as well as metastasis [51]. The profile of genetic mutations may change over time; the advantage of ctDNA is the possibility of repeating it during treatment. Sadakatsu Ikeda et al., 2018 point out that changes detected by ctDNA analysis were present in approximately 90% of HCC patients who had at least one potentially drug-sensitive lesion [8]. Matsumae T et al. performed ctDNA profiling using a panel for detecting mutations in 25 HCC-related cancer genes in 85 patients with non-surgical HCC (u-HCC) who received atezolizumab plus bevacizumab. CfDNA/ctDNA profiling has been shown to be a good biomarker for predicting the prognosis of patients with u-HCC treated with combined anti-PD-L1 and anti-VEGF immunotherapy [52].

Combined hepatocellular-cholangiocarcinoma (cHCC-CCA) is a primary liver cancer (PLC) and shows both hepatic and biliary differentiation [43,53]. Diagnosis of cHCC-CCA on the basis of tumor biopsy alone remains difficult [43,54], as it has characteristics of both HCC and CCA, so a tissue sample from an area similar to HCC or CCA may lead to misdiagnosis [43,55]. A histopathological evaluation of the biopsy or surgical material playsa key role in the diagnosis of cHCC-CCA. A lack of distinguishing features of cHCC-CCA imaging distinguishes the disease from HCC and CCA [43,56]. The cfDNA study of the bile of CCA patients showed high sensitivity and specificity in detecting single nucleotide changes, insertions and deletions (94.7 and 99.9%, respectively), and copy number variation (75.0 and 98.9%, respectively) [43,57]. Bile cfDNA has been demonstrated to be a reliable source of tumor genetic information, so bile fluid biopsy might be a promising approach for identifying CCAs. It has been proven that cHCC-CCA tumor tissue has a lower frequency of CTNNB1 mutations (only 6%) compared to HCC. Compared to CCAs, cHCC-CCAs had a significantly lower KRAS mutation rate (0%). As a result of the lack of mutations in both CTNNB1 and KRAS in cHCC-CCA is a unique feature [43,58]. Liquid biopsy might even be able to replace traditional tissue biopsy in the future by analyzing molecular changes in circulating tumor-derived molecules [43].

### 2.3. Prognostic Value

The detection of early HCC recurrence may be enhanced by ctDNA. Which are mainly caught by AFP levels and liver ultrasound, which has a sensitivity of only 63% [59,60]. In a Chinese study, it was confirmed that the majority of samples taken from patients at the time of suspected recurrence (97.4%, 75/77) taken at a time when the tumor could be confirmed by CT/MRI imaging methods were positive. In contrast, samples (100%, 42/42) from non-relapsed patients were, respectively, negative. In addition, in a few cases, positive ctDNA was detected in plasma samples before disease recurrence was seen on MRI, so cancer recurrence could be detected a median of 4.6 months in advance [51].

Zhu GQ et al. investigated the value of ctDNA in predicting early postoperative tumor recurrence and monitoring tumor burden in patients with HCC. They examined 41 patients who had undergone liver resection leading to a cure, with a confirmed radiological diagnosis. The genetic changes identified in the ctDNA were shown to be consistent with mutations present in the matched HCC tissue. Researchers have detected mutations of genes that were tested in ctDNA tissues and which are relevant in carcinogenesis: CTNNB1, TP53, NRAS, BRAF, and NFE2L2 [33]. A total number of 96 operatively treated patients diagnosed with primary HCC were enrolled in a study aiming to verify the prognostic value of ctDNA. It was found that ctDNA positivity in the immediate postoperative period was significantly associated with worse disease-free survival (DFS) and overall survival (OS). That indicates the clinical significance of ctDNA-based minimal residual disease (MRD) detection. Patients with positive postoperative ctDNA-AFP (H) subgroups and patients with positive ctDNA and Barcelona Clinic Liver Cancer (BCLC) staging C subgroups had the worst prognosis. Liquid biopsy markers such as postoperative ctDNA have a significant value in detecting high-risk recurrence patients [61].

Before surgery, ctDNA was detected in 63.4% of patients, while after radical liver surgery, only in 46% of patients. Serial ctDNA marking has been shown to reflect changes in real-time tumor mass well. There is a relationship between preoperative ctDNA and tumor size, differentiation, Microvascular Invasion, and early recurrence. In addition, preoperative detection of ctDNA has been shown to correlate with a high risk of tumor recurrence, confirming the role of ctDNA in monitoring disease progression. The most indicative time-point is probably postoperative, because, in most cases, the ctDNA is negative postoperatively, but in those who have prolonged positive results, the recurrence risk is high [33].

The results of a study involving 38 patients with HCC treated according to the trans-arterial chemoembolization (TACE) procedure suggest that cfDNA and ctDNA dynamics may become promising markers in assessing the effectiveness of treatment after the first TACE procedure. The changes in values between blood samples taken the day before TACE and values one month after TACE were +31.4% for cfDNA and 0% for ctDNA. As a result, they significantly predicted the worsening of the disease and were associated with worse PFS. In this study, the combined cfDNA and ctDNA scores were able to stratify patients into high- or low-risk groups for progressive disease (PD) one month after TACE, with a PD rate of 80.0% vs. 4.3% (*p* = 0.001) and a median progression-free survival of 1.3 vs. 10.3 months (*p* = 0.002) [40].

## 3. Comparison of ctDNA and Tissue NGS Results

NGS (next-generation sequencing) is a novel gene detection technology. It can detect many types of gene mutations in different types of chests and body fluid samples [11]. The ctDNA NGS differs from tissue NGS in that ctDNA shows genomic changes from excreted DNA from the primary tumor site and multiple metastatic sites, while tissue NGS shows changes present in the tissue fragment being assessed. Not every tumor releases DNA into the bloodstream at the same time, and the high vascularity of tumors increases the availability and speed of detecting changes in blood [62]. On the other hand, in patients with brain tumors, the blood–brain barrier may be an obstacle. One alternative procedure in the mentioned case may be a biopsy of the cerebrospinal fluid [63].

In the study by Sadakatsu Ikeda et al. in 26 patients with HCC in 6 cases, gene changes were found in both tissue NGS and ctDNA NGS, 14 changes were present only in tissue NGS, and another 18 changes were detected only in ctDNA NGS. The agreement in both studies for the most common mutations was 50% for TP53, 100% for *CTNNB1*, and 90% for *ARID1A*, respectively [8]. However, in a study of 213 patients with a diagnosis of gastrointestinal cancer, overall concordance was analyzed for 68 genes contained in tissue and liquid biopsy panels and found a concordance ratio of 96%, a McNemar P. value of 0.68, meaning ctDNA and tissue NGS is not significantly different [34]. Another study presented by An et al., 2019 showed a comparison between the ctDNA sequencing and tDNA (tumor DNA) results in a group of 26 patients. Out of 139 mutations detected in ctDNA, 69 (49.6%) could be validated in paired tumor DNA (tDNA). Another 70 mutations (50.4%) were only in plasma samples. At least one overlapping mutation could be detected in 23 patients (88.5%). Moreover, seven patients presented the same ctDNA mutations set in both ctDNA and matched tDNA. Nine presumptive driver genes for HCC, including *TP53*, *AXIN1*, *CTNNB1*, *CDKN2A*, *ARID1A*, *ARID2*, *SMARCA4*, *KEAP1*, and *NFE2L2*, were selected to explore their concordance between ctDNA and tDNA. A total of 37 driver events were identified in 23 patients (88.5%). Three patients did not present the presence of conventional driver events in both ctDNA and tDNA. Among driver events, 25 (67.6%) were shared in paired ctDNA and tDNA, 3 (8.1%) were found in plasma only, and 9 (24.3%) were exclusively in tissue material. Moreover, the concordance rate was higher among drivers than non-driver mutations (25/37, 67.6% versus 44/130, 33.8%, Chi-square *p*-value = 0.0002) [64].

Studies show that there is a strong correlation between ctDNA and tissue NGS. However, the advantage of ctDNA may be a higher rate of detection of MET changes, and they can detect primary tumor DNA shedding and MET changes, while tank assays detect biopsy localized changes. A blood test is less invasive compared to a biopsy, which may encourage repeat testing. The disadvantage of ctDNA testing, however, may be its lower sensitivity in detecting lesions that are easily identifiable by tissue testing, and not all disease sites may release DNA into the circulation, especially in early stages [62,65]. Both methods can independently detect neoplastic changes, emphasizing the clinical value in detecting and monitoring treatment and recurrence of the disease; it should be emphasized that they are complementary.

## 4. Circulating Marker Characteristics

The exact mechanisms of ctDNA release remain unknown. It is assumed that DNA fragments are disposed into the bloodstream by apoptosis and necrosis of cancer cells. There is no evidence regarding the variety of release among cancer clones. An experimental model in the study of Labgaa I. et al., 2021 aimed to assess the role of xenografts in monitoring ctDNA and CTC dynamics. The authors tried to check the possibility of DNA detection coming from different cell lines in xenografts, which would recapitulate intratumoral heterogeneity (ITH) as a result of clonal evolution. DNA specific and exclusive to each cell line mutations (*APOB* to Huh7 and *FGA* to HepG2) were targeted. It was found that the release of DNA fragments between the two clones varied. Further results showed that the concentration of ctDNA and the presence of tumor-specific mutations reflected tumor progression. The presented ITH model suggested a clone-dependent release of ctDNA. Differences in the release of DNA fragments were also caused by the treatment (sorafenib) [66].

The ctDNA contains information about tumor genome profiles, particularly single nucleotide variants (SNVs) and copy number variants (CNVs). High fractions of somatic SNVs and CNVs in plasma were detected in patients before surgery. On the other hand, after tumor resection, SNV and CNV were significantly reduced [51]. In a study by David Sefrioui and co-authors, it was observed that in patients with HCC after cytotoxic treatment, the release of ctDNA increased, which may indicate the process of apoptosis in cancer cells several hours after the administration of the drug [40].

Detection of ctDNA is different for individual cancers and depends on their stage of advancement. In a massive study involving 11,525 patients diagnosed with various cancers (HCC, n = 571), DNA was isolated from plasma. The sensitivity of ctDNA detection for HCC in stage I–III disease was 68.0%, 95% confidence interval was 62.6–73.4%, respectively. Stage IV ctDNA detection was similar for most tumors and was >70% [50].

## 5. Mutation in Hepatocellular Carcinoma 

Cancer-specific mutations have been reported in ctDNA from the peripheral blood of patients with HCC. These mutations include *CTNNB1*, *TERT*, *TP53*, *TMBIM6* and *MLH1* [3,67,68,69,70,71]. They are described in detail below. Other frequently detected genes in HCC were: *EGRF*, *MYC*, *CDK6*, *TMEM141*, *UBB*, and *ADGRV1*, etc. [3,72]. Moreover, there were no patients with the same ctDNA-derived somatic mutation pattern [72]. The less common genetic mutations and the characteristics associated with them are included in Table 1.

### 5.1. CTNNB1

One of the most prevalent genetic alterations in HCC is the mutation of the *CTNNB1* gene, which can appear at any moment during the evolution of the HCC [88,89]. In the Jian Gao study, mutations in the *CTNNB1* gene were diagnosed in 15% of patients with HBV-related HCC [3]. 

One of the most frequent genetic events in HCC is a gain of function (GOF) mutation of *CTNNB1*, and it could be found in 15 to 30% of human HCCs [90,91]. It was found that proteins that are involved in various metabolic activities, such as drug and amino acid metabolisms, glycolysis, and gluconeogenesis, are enriched in *CTNNB1* mutant tumors [92,93]. *CTNNB1* mutation usually occurs at a later stage of HCC progression, and there is still no adequate study of its mechanism [92].

A metabolic morphotype of *CTNNB1*-mutated HCC is specific, andit is often cholestatic and sometimes steatotic [89,92,94]. It was shown that HCCs with GOF (gain of function) *CTNNB1* mutations display a particular, well-differentiated phenotype, infrequent microvascular invasion, low α-fetoprotein (AFP) levels, and better outcomes [90]. 

The oncogenic Wnt/β-catenin pathway, activated by the mutated *CTNNB1*, plays an important role in the metabolic regulation in the liver [75,92]. Studies have shown the presence of the Wnt/β-catenin pathway for ex. *CTNNB1* gene mutation indicates a weaker response to immunotherapy [95]. However, ORR (overall response rate), DCR (disease control rate), PFS (progression-free survival), and OS (overall survival) have been shown to be similar for both *CTNNB1* mutation and *CTNNB1* mutation absence [52].

One of the aims of a study by Oversoe, S.K. et al., 2021 was to evaluate the concordance rates between plasma and tissue samples. Results of the study showed that analysis of ctDNA can reveal tumor mutations that are not apparent in single tumor biopsies. The detection rate of *CTNNB1* mutation in HCC patients may be increased by combining those two methods. Serial analysis of ctDNA enables monitoring of tumor mutation profiles and may help with creating personalized therapeutic strategies [96].

### 5.2. TERT 

The presence of *TERT* mutations has been shown to be associated with poor prognosis in patients with different types of cancer [97]. The *TERT* gene is involved in the telomeric cycle and is altered in approximately 60% of HCC cases. These *TERT* changes consist of two hotspot mutations within the gene promoter [40]. 

Mutations in the *TERT* gene may be a potential screening target for both early stage and advanced HCC. In a study by Zhang Y. and others, *TERT* promoter SNVs were most commonly found among HCC patients with early stage cancer in 22.3% of cases [50]. The aim of the study by Hirai et al., 2021 was to detect *TERT* mutations in the ctDNA of patients with advanced HCC. The study group consisted of 130 patients: 86 were treated with systemic chemotherapy (sorafenib and/or lenvatinib) and 44 with transcatheter arterial chemoembolization. The *TERT* promoter mutations in ctDNA were detected in 71 patients (54.6%) [98]. The presence of *TERT* mutations, as well as its high fractional abundance (≥1%), was associated with shorter overall survival than in patients without them [52,98].

The study by Ge, Z. et al. aimed to detect the oncogenic mutations in paired circulating tumor DNA and circulating tumor cells in patients with HCC. It was found that the correlations between liquid biopsy results and clinicopathologic parameters vary between ctDNA status and CTC count. *TERT* C228T (or ctDNA positivity) correlated with macrovascular invasion (MVI) as found in CT/MRI imaging. Such correlation was not observed for CTCs. Results of the linear regression analysis showed a positive correlation between ctDNA VAF and the size of the largest tumor as well as AFP level. Similarly, this correlation with AFP level and tumor diameter was observed for *TERT* C228T VAF. However, CTC count did not correlate with the largest tumor diameter or AFP [13]. 

### 5.3. TP53

Wild-type *TP53* is involved in cell cycle regulation and apoptosis following DNA damage [99]. If the *TP53* gene is mutated, DNA-damaged cells can escape apoptosis and become cancerous. Additionally, mutant *TP53* proteins lose their wild-type functions and accumulate in the nucleus. There is a strong correlation between this accumulation and malignant tumors [100]. 

In a study that isolated ctDNA from 26 patients with advanced HCC, the most common (61.5%) mutations concerned the *TP53* gene. They observed the highest frequency of mutant ctDNA alleles in the *TP53* mutation at 12%. This is an important fact because *TP53* overexpression is a factor in poor prognosis in patients with HCC [8]. In a Chinese study on 26 HCC patients, the NGS method was used for ctDNA profiling. Accordingly, 96.2% of patients with HCC had ctDNA mutations that could be validated through matched tumor tissue testing. A potential screening marker and the most common mutation was *TP53* R249S. Since the detection sensitivity of this mutation in ctDNA was 83.3%. Several cfDNA parameters were linearly related to tumor diameter. In the future, the use of ctDNA for postoperative HCC observation may be attempted [64]. 

TP53 mutations may be involved in the regulation of HBV-associated HCC. In Jian Gao’s study, mutations in the *TP53* gene were present in 38% of HCC patients due to HBV infection, while in HCC and HCV patients, it was present in 20% of patients [3]. In the group of 96 patients in the study by Ye, K. et al., 2022 it was observed that mutations of *TP53* were associated with relapses (*p* = 0.0106). It may be closely associated with cancer due to HBV infection and exposure to aflatoxin [61,101,102].

### 5.4. TMBIM6

BAX is a member of the Bcl-2 family of pro-apoptotic proteins [103]. It is involved in both intrinsic and extrinsic apoptotic signaling and concentrates mainly in the cytosol. The intrinsic way can be triggered by oncogenic stress, chemotherapeutic agents or metabolic stress, while the extrinsic pathway (death receptor pathway) activation depends on the interactions between death receptors and their ligands of the tumor necrosis factor (TNF) family [104]. Apoptosis is dependent on the pro-apoptotic BCL-2 proteins BAX and BAK can be induced by cytotoxic cell stress. BAX protein activity is restricted by the constant shift from the mitochondria to the cytosol. Changes in the BCL-2 regulation and the appearance of antiapoptotic effects are one of the most remarkable changes in HCC development. Abnormalities regarding BAX, such as inactivated mutations or down-regulations, can affect the ratio of BAX/BCL-2, resulting in resistance to cell death [104]. 

Funk et al., 2022 presented a study on the effects of *TMBIM6* localization equilibrium changes in hepatocarcinogenesis. There are two groups of HCC regarding *TMBIM6* protection status. The first group is BAX protected tumors, which experience elevated levels of oxidative stress and cytosolic concentration of *TMBIM6*. Those cells are prone to cellular stress resulting in DNA damage and further activation of DNA repair mechanisms. The second group is *TMBIM6* non-protected tumors. Those tumor cells present mitochondrial dysfunction and higher proliferative capacity. Differences between those groups are followed by various responses to treatment. *TMBIM6* protected tumors present increased sensitivity to PARP inhibitors, while *TMBIM6* non-protected HCC may respond better to classical chemotherapy [105]. In a study by Alunni-Fabbroni, M. et al., 2019 in 13 male HCC patients treated with sorafenib, one of the most frequently detected mutant genes was *TMBIM6* (69.2% of patients). *TMBIM6* may cause low efficacy of sorafenib therapy by inhibiting the apoptosis pathway induced by this drug. However, this study did not show a significant correlation between *TMBIM6* and survival time. Researchers found a significant correlation between the presence of *TMBIM6* variants and portal vein invasion [106].

### 5.5. MLH1

The *MLH1* gene plays a key role in the repair of DNA strand breaks, and it has been shown that *MLH1* is immediately recruited to sites of DNA strand breaks caused by DNA-damaging agents [107]. It also has a significant role in the repair of mismatched bases in the DNA strand [108]. The *MLH1* polymorphism has been detected in colorectal cancer, endometrial cancer, prostate cancer, pancreatic cancer, head and neck squamous cell carcinoma, and oral squamous cell carcinoma [109,110,111,112,113]. However, there are only a few studies that support an association between *MLH1* polymorphisms and HCC.

In one Korean study, 107 HCC patients were assessed for ctDNA amounts. The patients were divided into two groups according to the median concentration of recovered ctDNA: low (<5.77 ng/mL) and high (≥5.77 ng/mL). There was a significant increase in the proportion of patients with high ctDNA levels with the progression of the stage of HCC and those with vascular invasion. Patients with *MLH1* or *NPM1* mutations were found to have poorer Overall Survival compared to non-mutated patients. The SNV*MLH1* was proven to be associated with BCLC (Barcelona Clinic Liver Cancer) (*p* = 0.025). After analyzing subgroups of patients with highly advanced HCC (BCLC stage C/D or modified Union for International Cancer Control stage IV), it turned out that patients with *MLH1* SNV detected in ctDNA had lower survival rates than those without this mutation. The absence of *MLH1* mutations correlated with low AFP levels and was associated with the best overall survival (*p* = 0.005) [113]. In a study by Xiao Nian Zhu et al., 2017 including 436 patients with HCC, the presence of single nucleotide polymorphism (SNP) *MLH1*, rs1800734, was shown to correlate with tumor size, stage, and AFP level of HCC patients (*p* < 0.05). However, between the other three *MLH1* SNPs: rs10849, rs3774343, and rs1540354, no such associations were observed. Each of these *MLH1* polymorphisms interacted with HBV infection, alcohol consumption, and smoking. The *MLH1* rs1800734 SNP had an interaction with the other SNPs rs10849, rs3774343, and rs1540354, these SNP-SNP interactions resulted in a higher incidence of HCC, but no association was detected between the presence of *MLH1* SNPs and a positive family history of HCC [114]. These reports suggest that *MLH1* mutations may have a significant role in the development and prognosis of HCC patients.

### 5.6. DNA Methylation Changes

The abnormal methylation of DNA has been considered to be one of the key factors involved in the development and progression of cancer [115]. It has been demonstrated that global hypomethylation is associated with genome instability and loss of imprinting, resulting in increased cancer risk [116]. During the transformation from normal to cancerous tissue, DNA hypermethylation occurs at the promoter regions of tumor suppressor genes [117,118]. Almost half of the studies on biomarkers in HCC have focused on gene methylation in the last five years. In ctDNA, methylation biomarkers can be classified into three categories: the number of methylations, the expression of methylations, and the detection of 5-hydroxymethylcytosin [119].

In a study of 227 tissue samples from HCC patients that assessed DNA methylation profiles, HCC tissues showed a much greater variation in DNA methylation compared to non-malignant tissue. Additionally, the methylome DNA of adjacent cirrhotic non-cancerous tissue from HCC captures prognostic features in HCC patients. Researchers detected promoter hypermethylation in four genes (*TSPYL5*, *KCNA3*, *LDHB*, and *SPINT2*) that are involved in the regulation of *TP53*, cAMP, serine protease, and NADH, and a corresponding decrease in gene expression in early HCC [120]. HCC ctDNA samples contained a wide range of hypermethylated gene sites, including *THY1*, *DBX1*, *GPBAR1*, *CDKN2A*, *VIM*, *FBLN1*, *RGS10*, *RUNX*, *MT1M*, *MT1G*, and *RGS10* [121]. In an interesting finding, researchers found that hypomethylation in HBV integration regions is associated with a higher sensitivity for diagnosing HCC [122]. Na Hu et al., 2017 in their study of 80 patients with HBV-related HCC, showed hypomethylation of the *UBE2Q1* gene in the sera of HBV-associated HCC patients and also noted a negative correlation between *UBE2Q1* gene methylation and the TNM stage of the tumor [123].

## 6. Limitations of ctDNA

Although liquid biopsy seems to be a promising tool in HCC management with its non-invasiveness and possible repetition of tumor biology examinations, it comes with limitations. There is still a fraction of tumor-derived mutations that do not appear in ctDNA profiling, mostly subclonal mutations that have inferior allele frequencies in tissue samples [124]. The molecular profiles of tumors differ due to their heterogeneity. Thus, in a person without confirmed cancer, it can be difficult to determine whether a nucleic acid signature is a ctDNA molecule, especially in the early stages, where a tumor may show a small subset of mutations. Even in a plasma sample with the same tumor genotype, intratumoral heterogeneity may lead to differences in ctDNA mutation concentrations [125]. It is common for studies to have a limited sample size as one of their limitations. The challenging aspect of a noninvasive blood test is determining the sample size needed to adequately capture heterogeneous genomic aberrations in tumors with extensive heterogeneity [6,124,126]. The widespread clinical application of liquid biopsy is not yet on the horizon due to both the related cost and technology. Moreover, the majority of data supporting its utility derives from proof-of-concept studies, mainly retrospective, and not validated by different researchers, and there needs to be a standardized assay protocol with high sensitivity and specificity [127,128].

## 7. Conclusions

Liquid biopsy might decrease the need for invasive diagnostic procedures in patients with HCC. Currently, known markers need further research on their specificity in detecting HCC and application in disease monitoring. Molecular diagnostics enables the creation of new therapeutic approaches. ctDNA provides detailed and dynamic tumor data, making it a hopeful marker for both prognostic and diagnostic use in patients with hepatocellular carcinoma; it is a promising material for planning a personalized treatment. Further extended studies on the use of ctDNA in patients with HCC are needed; currently, this technique is quite time-consuming. Although costs are currently high, they decrease over time, and the usefulness of ctDNA in liquid biopsy may significantly change the clinical management of HCC. The replacement of currently used tools in the management of HCC patients by liquid biopsy biomarkers is unrealistic, but they will likely be integrated into the process, providing a stronger predictive power.

## Figures and Tables

**Table 1:** The less common genetic mutations detected in the ctDNA, and the characteristics associated with them.

Gene	Classification	Type OF Change	Role	Clinical Implications	References
*CDKN2A*	Suppressor gene	Homozygous deletions in chromosome 9	Regulation of immune cell functionality; encodes CDKN2A protein that inhibits the G1 to S transition in cell cycles through binding to cyclin-dependent kinases	Poorer overall survival and disease-free survival; prognostic biomarker correlation with immune infiltrates in HCC	[73,74,75]
*ARID1A*	Suppressor gene	Inactivating mutation	Remodeling of chromatin activity by binding transcriptional co-activator/co-repressor complexes	Deceleration HCC progression and post-treatment tumor recurrence; promotes tumor initiation	[76,77]
*ARID2*	Suppressor gene	Inactivating mutation	Downregulate mesenchymal markers, upregulate epithelial markers involved in the disruption of chromatin regulatory processes	Negative correlation with organ metastasis, pathological grade and positive association with survival of HCC patients	[78,79]
*RASSF1A*	Oncogene	Methylation	Suppresses PI3K-AKT-mTOR pathway to promote autophagy initiation through Hippo pathway regulatory protein MST1; apoptosis cell cycle, microtubule stability, cell adhesion and migration	Promising biomarker for the diagnosis of HCC in tissue and blood	[80,81,82,83,84,85]
*SEPTIN9*	Suppressor gene	Methylation	Role in the growth of lipid droplets, cytokinesis	Plasma mSEPT9 positively correlated with HCC malignancy, detection of early stage HCC	[86,87]

## Data Availability

No new data were created or analyzed in this study. Data sharing is not applicable to this article.
